# Revealing the Intellectual Structure and Evolution of Digital Addiction Research: An Integrated Bibliometric and Science Mapping Approach

**DOI:** 10.3390/ijerph192214883

**Published:** 2022-11-12

**Authors:** Turgut Karakose, Tijen Tülübaş, Stamatios Papadakis

**Affiliations:** 1 Faculty of Education, Kutahya Dumlupinar University, 43100 Kutahya, Turkey; 2 Department of Education, University of Crete, 74100 Rethymno, Greece

**Keywords:** digital addiction, Internet addiction, social media addiction, smartphone addiction, game addiction, technology addiction, bibliometric analysis, science mapping, SciMAT

## Abstract

The current study uses “digital addiction” as an umbrella term to refer to addiction to the Internet, social media, smartphones, digital devices, video games, or similar digital technologies. The study aims to investigate the scientific landscape of the digital addiction research field through combining bibliometric and science mapping analysis and to facilitate insight into the development and evolution of this knowledge base. Data for the analysis were extracted from the Scopus database, which covers a wide range of journal articles, with 429 articles addressing digital addiction included in the analysis. The science mapping analysis was performed over three consecutive time periods using SciMAT software to observe the thematic evolution. The results indicated that addictive behavior was the most significant theme across the three time periods. Cross-sectional studies addressing the risk factors or outcomes of addiction among adults and different sexes were prevalent during the first period (1997–2012), while interest moved to the addictive behavior of adolescents and students during the second period (2013–2017). Research during the third period (2018–2022) was driven by smartphone and social media addiction, and resilience as a preventive factor garnered more research interest than previously studied risk factors, which may indicate a perspective change by researchers. Implications are suggested for future investigations of digital addiction.

## 1. Introduction

Recent decades have witnessed a breakthrough in communication and information technologies, and many types of digital media (i.e., mediums of broadcasting any digitized information through a screen or/and a speaker for the purposes of communication, entertainment, or dissemination of information) have become an indispensable part of everyday life [1]. The adoption of digital technologies—namely the electronic tools; devices; systems; and resources used to generate, store, or process data such as the Internet and smartphones—have influenced people and societies in significant ways [2]. This process of digitalization is considered to be as revolutionary as the invention of the wheel, the steam engine, or even electricity [3], which are all examples of things once believed to be impossible having before they became a reality in life [4]. Yet, despite offering a wide range of possibilities and significant convenience to almost every aspect of modern-day life, there is a growing consensus that digital technologies have also created certain serious challenges and adverse effects on human relations, as well as both mental and physical health [5,6]. It is increasingly considered that usage of smartphones, social media, video games, and other such innovations can be as addictive as tobacco smoking, taking nonprescribed drugs or illicit substances, or gambling [7,8]. Some even claim that digital media is a powerful “new drug” that presents a high risk of addiction [9]. Indeed, as Meng et al. [10] recently stated, the global prevalence of smartphone addiction has reached nearly 27%, whilst Internet addiction has reached almost 15%, and game addiction is at 6%, indicating the potential addictive nature of using digital technologies.

### 1.1. Digital Addiction

Behavioral addictions due to the overuse of digital technologies have become of serious concern to both scholars and practitioners since the early 1990s, although the phenomenon was then recognized only as Internet addiction but covered addiction to any kind of online activity [11]. However, with the introduction of various digital devices (e.g., smartphone, tablet personal computers, and gaming consoles) and affiliated digital activities (e.g., social media, video games, and cybergames), the scope of such addictions has consolidated into a much broader term—“digital addiction” [8,12].

Digital addiction is an overarching term that refers to the compulsive, prolonged, and uncontrollable use of any digital device or media with deleterious effects on the psychological or physical health of users [10,13] and thus incorporates addiction to the Internet, social media, smartphones, video games, or any other related digital technology devices [6,8]. Digital addiction does not necessarily entail the use of the Internet, since digital devices can also be used in an addictive manner without requiring an Internet connection (e.g., addiction to offline gaming, excessive use of offline mobile apps, etc.) [12,14]. However, the Internet, as a channel used to access digital material, can facilitate addiction through enabling users to instantly access whatever content they want from virtually any place and at any time [9].

Although digital addiction is a relatively new term in the scientific world, scholars have defined its core features based on those of addictive behavior. Some scholars contend that digital addiction occurs through habit formation and self-control problems, just as seen in other behavioral addictions such as gambling or substance abuse [5,15]. The combination of these two forces often results in preoccupation with the digital media/device, repeated unsuccessful attempts to stop or reduce usage, greater utilization than expected or warranted, deleterious effects on school or work life, damage caused to relationships or social life, and even lying about the level of actual usage, all of which characterize typical addictive behaviors [9,16]. In other words, digital addiction “suggest[s] an increasing trend of compulsive behavior amongst users of technological devices, recognizing that over-exposure to and over-use of technology can result in a dependence on digital devices, leading to behavioral symptoms similar to any addictive disorder” [4] (p. 18). However, in the case of digital addiction, the instant availability of the content being craved through the digital medium, without necessarily requiring any intermediary dealer, makes it an even more complex syndrome [9].

Digital addiction has now become a global concern, as research increasingly reveals its serious consequences on both the physical and psychological well-being of users, with impairments seen in social life (e.g., social isolation, neglect of social activities, and social conflict) [17]; emotional impairment (e.g., anxiety, depression, and restlessness) [18,19]; physical impairment (e.g., poor eyesight, backache, and weight gain/loss) [20,21]; cognitive disorders (e.g., poor concentration and changes in brain function) [22,23]; and decreased performance and productivity at school or work [24,25]. Thus, digital addiction has attracted significant interest from both scholars and practitioners across a variety of fields such as psychology, sociology, neuroscience, and education.

### 1.2. The Purpose of the Study

The influence of technological revolution and digitalization on people and society have urged systematic studies to develop a broader conceptualization of digital addiction and to provide empirical evidence to support its detection, prevention, and treatment [10]. Incremental interest in digital addiction has aggregated a significant collection of intellectual contributions to this research field during the last two decades [11]. Nonetheless, to the researchers’ knowledge, the intellectual structure and the thematic evolution of this field have yet to be investigated. In order to address this gap in the literature, the current study aims to investigate the scientific landscape of digital addiction as a research field through combining bibliometric and science mapping analysis and facilitate insight into the development and evolution of this knowledge base. The study particularly aims to reveal (1) the volume and growth trajectory of scholarships on digital addiction, (2) journals and authors that have evidenced the greatest citation impact on digital addiction, (3) the intellectual structure and evolution of the digital addiction knowledge base, and (4) the themes that have attracted the most attention on digital addiction.

## 2. Materials and Methods

### 2.1. Study Design

The current study combines the bibliometric performance analysis and scientific mapping methods to determine the bibliometric performance of the digital addiction research field, to reveal its dynamic and structural features, and to delineate its cognitive and conceptual architecture [26,27].

### 2.2. Data Search and Identification

**Figure 1:**
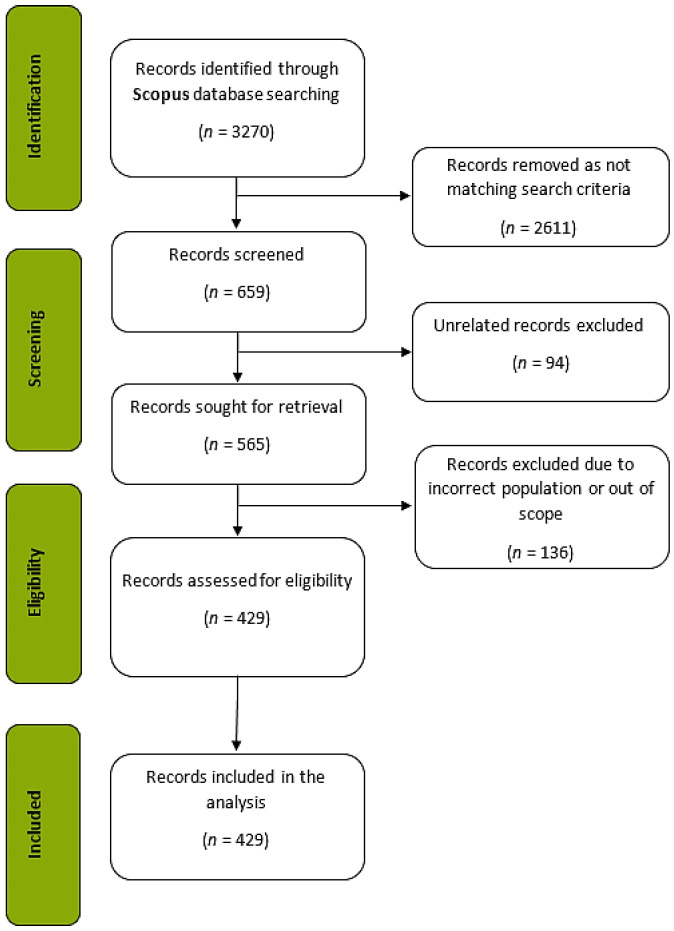
PRISMA flow diagram.

Bibliometric studies often use online databases such as Web of Science (WoS), PubMed, and Scopus as data sources. Scopus is defined as one of the optimum databases used for bibliometrics [28], since it lists more journals than WoS and covers almost all publications listed in the WoS database [29]. This helps to reduce the risk of missing documents published that would be valuable to the analysis. Therefore, data for the current study were searched for and extracted from the Scopus database. Data collection and analysis were conducted following a three-step procedure: (1) searching and defining data, (2) extracting and cleaning data, and (3) analyzing data [30]. The selection process of the 429 articles included in the final analysis is reported according to PRISMA (Preferred Reporting Items for Systematic Reviews and Meta-Analyses) guidance [31], as illustrated in Figure 1.

The following inclusion/exclusion criteria in Table 1 were applied to select the data for analysis.

The following keyword string was used to perform a keyword search on the Scopus database on 9 July 2022:

*TITLE (“digital addiction” OR “internet addiction” OR “social media addiction” OR “smartphone addiction” OR “mobile phone addiction” OR “digital media addiction” OR “game addiction” OR “virtual addiction” OR “technology addiction” OR “computer game addiction” OR “gaming addiction” OR “mobile addiction” OR “online shopping addiction” OR “cybersex addiction” OR “gadget addiction” OR “mobile apps addiction” OR “sms addiction” OR “selfie addiction”)*

The keywords were selected after an in-depth review of the relevant literature and the approval of three field experts. A total of 3270 documents were listed after the initial search. During the data selection, all three authors of the manuscript individually scanned through the titles and keywords of these 3270 articles yielded during the first cycle of search. Next, they skimmed through the abstracts of selected 659 articles. Finally, they organized a peer-debriefing to discuss over which articles to include in or exclude from the analysis and agreed upon 565 articles. The abstracts of the remaining 565 articles were read, and 136 were excluded, because they were incorrectly populated and/or were considered to be outside the scope. The authors also conferred with two other scholars when they could not reach an agreement. In the end, a total of 429 research articles were selected to be included in the study.

### 2.3. Data Extraction and Analysis

The bibliometric data of each article selected for inclusion in the analysis (title, author names, keywords, abstract, citations, publication date, and journal name, plus other information such as country of origin) were transferred to SciMAT (Science Mapping Analysis Tool) software-version 1.1.04. Using SciMAT, keywords found to be representative of the same concept (e.g., adolescent and adolescents and self control and self-control) were manually grouped in order to increase the quality of the thematic analysis [32,33].

First, a bibliometric performance analysis was performed to determine the temporal distribution of related articles, the accumulated number of articles, the average number of citations per article, the most cited articles, the most cited authors, and the most influential journals and countries [34]. Then, the SciMAT software tool was used to perform a science mapping analysis [32], which is a useful method to determine the conceptual structure, evolution, and research trajectory of a particular research field and delineates the structural and dynamic aspects of scientific research in that particular field of study [26,35]. SciMAT is considered a particularly powerful tool, since it allows for the analysis and monitoring of a research field’s evolution over sequential time periods [32,36,37,38]. In the current study, four steps were followed to conduct a science mapping analysis using SciMAT software [26,32,35,39,40,41]: (i) identification of digital addiction research themes, (ii) visualization of research themes and thematic network, (iii) identification of thematic areas, and (iv) performance analysis.

*(i) Identification of digital addiction research themes:* A standardized network of co-occurring keywords was formed based on the keywords extracted from the retrieved articles. Next, in order to identify the themes of the published research, a clustering algorithm was applied to the normalized network of cooccurring keywords, with closely related keywords making up each cluster or theme. Thus, the conceptual subfields of digital addiction as a research field were identified, and their thematic evolution was revealed.

*(ii) Visualization of research themes and thematic network:* A graphical representation of the identified research themes was created using two different tools, strategic diagram and thematic network. The current study employed a clustering algorithm to identify and illustrate the research themes. The themes emerged from the cluster-based analysis were presented in a four-quadrant, two-dimensional strategic diagram based on the density (*y*-axis) and centrality (*x*-axis) values. Density represents the capacity of themes in a research field to persist and develop over time. In addition, density relates to the internal relations of the themes. Themes with increased relationships among themselves shift upwards towards the top of the strategic diagram. Density is mathematically formulated as “*d* = 100 (Σ*eij*/*w*)”, where “*i*” and “*j*” represent the keywords of the theme, and “*w*” represents the number of keywords in the theme. Centrality measures the degree to which a cluster interacts with other clusters or the strength of its relationship. In other words, centrality relates to the external relations of a theme. Therefore, as the relationship of a theme with other themes increases, the themes shift to the right-hand side of the strategic diagram. Centrality is mathematically formulated as “*c* = 10 × Σ*ekh*”, where “*k*” represents a keyword belonging to any one theme, and “*h*” represents a keyword belonging to another theme. Centrality reveals that a cluster or network is an important crossing point and has therefore a critical role in highlighting and helping to understand the relationship between themes. Density, on the other hand, measures the internal strength of the relationship—that is, the strength of the relationship between keywords within a theme. Centrality and density values enable a research field to be represented in a strategic biaxial diagram incorporating four different categories.

**Figure 2:**
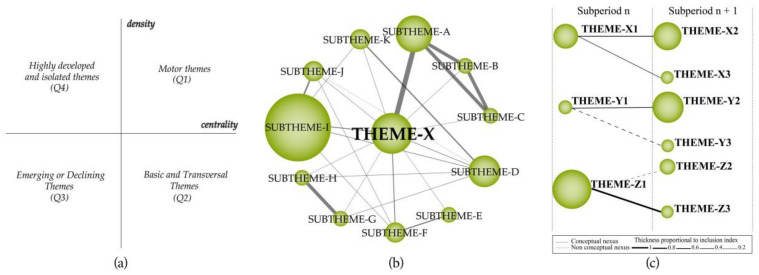
(**a**) Strategic diagram, (**b**) thematic network structure, and (**c**) thematic evolution structure [32].

Accordingly, in the current study, the research themes revealed through conceptual science mapping analysis based on a network of cooccurring keywords are presented in a strategic diagram (see Figure 2a) and is comprised of four groupings [32]: *(a) motor themes (Q1):* high centrality and density (themes related to the development and structuring of the research field, i.e., they show high progress and are the most significant themes for that field of research; *(b) basic and transversal themes (Q2):* high centrality and low density (themes not that well-developed but related to the research field but still tend to be motor themes due to their high centrality); *(c) emerging or declining themes (Q3):* low centrality and density (themes emerging or already disappeared, which can be determined through an in-depth qualitative analysis); and *(d) highly developed and isolated themes (Q4):* low centrality and high density (despite being highly specialized and environmental themes, they may be no longer deemed important or lack the appropriate background due to newly emerged concepts in the field).

The thematic network structure (see Figure 2b) was used to present how strategic themes emerge alongside other subthemes related to the research field, with each thematic network tagged according to the most important (most central) keyword in the associated theme. The keywords are interconnected, with the volume of the spheres being proportional to the number of articles corresponding to each keyword. The size of the circles in the thematic networks correspond to the number of articles, whilst the thickness of the lines shows the strength of their relationship.

The evolution of research themes, time periods, origins, and interrelationships developed over different time periods are presented in the thematic evolution map (see Figure 2c). A theme may belong to a different thematic area or may not be a continuation of any other theme depending on their interrelationship. Solid lines on the thematic map indicate that the same keywords as the theme names are shared between the themes, whilst the dashed lines indicate that common words are shared apart from the theme labels. The thickness of the lines varies according to the degree of the relationship, whilst the size of the circles corresponds to the number of articles.

*(iii) Identification of thematic areas:* The data were divided into three consecutive time periods so as to analyze the evolution of the digital addiction research field. The conceptual links between themes from the different time periods were explored using an inclusion index, which is based on the equation: Ii = #(U ∩ V)/min(#U, #V) [42,43]. The thematic evolution map was created through forming conceptual links (common keywords) from the U theme to the V theme. A thematic connection between the U theme and the V theme shows that both themes have common elements, as well as reflecting their evolution over time. As the number of common keywords between periodical clusters increases, their evolution becomes more evident.

*(iv) Performance analysis:* The number of articles, total citations, and different h-index values [44,45] were used as bibliometric indicators in order to measure the contribution (scientific impact) of each thematic area that emerged from the analysis to the whole digital addiction research field.

An analysis made over different time periods is suggested to save the data from uniformity and is used to obtain comparative results [33]. Hence, the articles selected for analysis were evaluated over three consecutive time periods that were formed based on the number of articles published over the whole period of analysis (1997–2022). Period 1 was comprised of the years 1997–2012 due to the lower number of articles published during the earlier years, whilst Period 2 was comprised of the years 2013–2017, and Period 3 was comprised of the years 2018–2022.

## 3. Results

### 3.1. Overall Bibliometric Analysis

A performance-based bibliometric analysis was first conducted in order to evaluate the digital addiction research field. Using the bibliometric indicators, the distributions of the articles according to their year of publication, the accumulated number of articles, numbers of citations per article, most productive/cited authors, most productive/cited journals, and most productive countries were determined [45].

#### 3.1.1. Publication Trends

**Figure 3:**
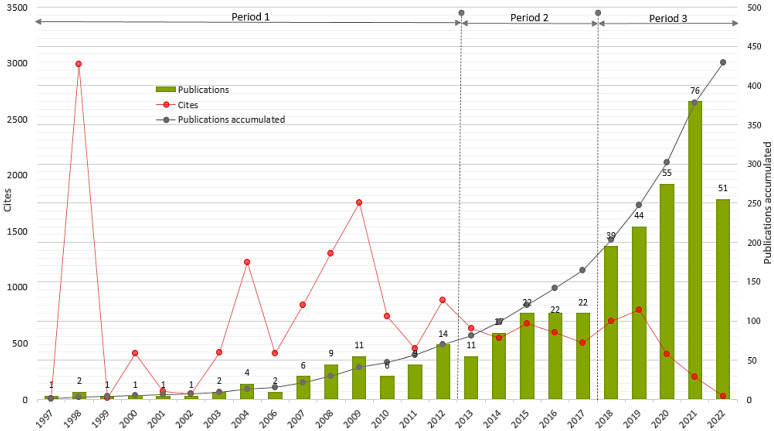
Distribution of publications and citations by year (1997–2022).

The distribution of the 429 articles included in the current study by year of publication, the accumulated number of articles, and a graphical representation of the average number of citations per article are presented in Figure 3. The *red line* illustrates the yearly citation rates, whilst the *grey line* refers to the number of publications accumulated per year, and the *green columns* show the annual distribution of the articles.

As can be seen in Figure 3, research interest in the digital addiction research field increased incrementally from 1997 to 2022, and in general, the number of articles increased during the last years. As can be clearly seen, the number of articles published on digital addiction peaked during 2020 and 2021, which may indicate scholars’ interest in the prevailing influence of increased exposure to digital media during the COVID-19 pandemic. The growth of research into digital addiction also has parallels with the advancement and accelerating prevalence of digital technologies in the current age.

#### 3.1.2. Most Influential Authors

The top 10 most productive research scientists who contributed to the digital addiction literature are listed in Table 2 based on the total number of citations received by their published articles. Within the scope of the 429 articles analyzed, the number of authors that contributed to the articles totaled 1177, with some authors also having been involved in more than one of the published studies.

As can be seen in Table 2, Young, one of the first authors to address Internet addiction and who devised the first questionnaire to measure the phenomenon in 1998, contributed to the field with the highest citation rate (*n* = 3219). Ko followed Young with the highest number of articles published (*n* = 7) and second-highest number of citations (*n* = 1072).

#### 3.1.3. Most Influential Journals

The top 10 journals that published the highest number of articles on digital addiction between 1997 and 2022 are listed in Table 3, based on the total number of articles published.

As shown in Table 3, many of the articles on digital addiction were published in the *Cyberpsychology, Behavior, and Social Networking* journal; previously known as *Cyberpsychology and Behavior*, which emerged as one of the most influential journals in the field. When the publications of these two journal names are considered cumulatively, they represent the highest number of articles published in the digital addiction research field, followed by *Children and Youth Services* and *Turkish Online Journal of Educational Technology* with 25 and 10 publications, respectively.

#### 3.1.4. Most Cited Articles

The top 10 articles that received the highest number of citations among the 429 articles included in the analysis are listed in Table 4.

As can be seen in Table 4, the most influential article published within the digital addiction field of research was one of the first publications in the field with a focus on Internet addiction. The second article with the highest number of citations, on the other hand, presented a new scale on gaming addiction, which is widely used to measure levels of gaming addiction. It is also notable, however, that the articles published between 2010 and 2022 have, to date, received much less interest compared to those published prior to 2010.

#### 3.1.5. Most Productive Countries

The 10 most productive countries that contributed to the digital addiction research field are listed in Table 5.

As can be seen from Table 5, Turkey emerged as the country having published the most articles on digital addiction. As previously mentioned, the *Turkish Online Journal of Educational Technology* was among the top three most productive journals on digital addiction, which correlates to Turkey’s leading position in Table 5. After Turkey, China, the United States, and South Korea are shown to hold pioneering positions in the advancement of digital technologies. It is also noteworthy that Table 5 presents a global view of the interest and contribution to the digital addiction field of research.

### 3.2. Science Mapping and Performance Analysis

In this section, the articles obtained from the Scopus database were analyzed using SciMAT software, and the findings are reported as: (i) period-based thematic analyses, (ii) overlapping graph analyses, and (iii) evolution map analyses.

#### 3.2.1. Scientific Evolution Structure

##### Period 1 (1997–2012)

**Figure 4:**
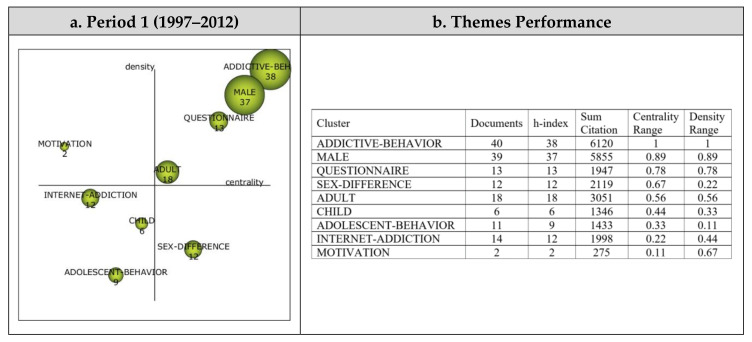
Period 1: (**a**) Strategic diagram and (**b**) performance analysis.

Nine themes emerged from the analysis of the 70 articles reviewed in the scope of Period 1. The strategic diagram and the performance measures for the themes that emerged during the first period are presented in Figure 4.

A total of nine main themes emerged during the first period (1997–2012). Addictive Behavior, Male, Questionnaire, and Adult were found to be the motor themes, which contributed the most to the development of the digital addiction research field. The Motivation theme emerged as a highly advanced and isolated theme, which is defined as having a strong basis on its own but not providing an appropriate or significant background for this field of research. The Internet Addiction, Adolescent Behavior, and Child themes were shown to be among the emerging/declining themes, which may have emerged during the period yet remained as weak or disappearing themes. The Sex Difference theme was included as a basic and transversal theme, which means that, despite being related to the research field being studied, it had not developed sufficiently during the time period. The theme shown to be of the most significant importance during Period 1 was Addictive Behavior, which was a motor theme represented by 40 published articles.

**Figure 5:**
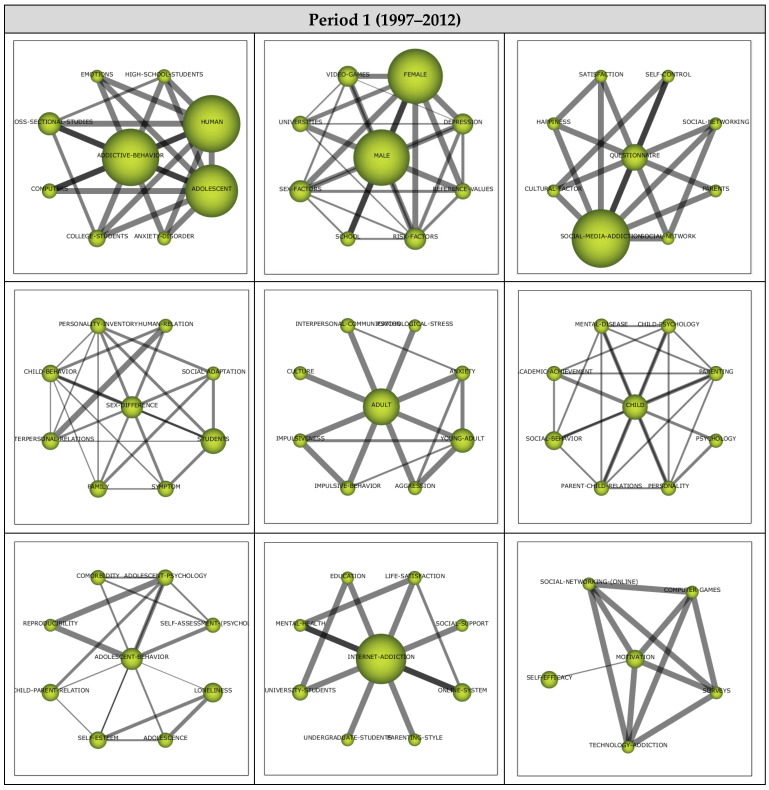
Period 1: Thematic network structures.

Cluster networks were examined (see Figure 5) in order to determine the related subthemes that emerged during the first period (1997–2012). Accordingly, it was determined that the main theme of Addictive Behavior (1, 1) was strongly related to the subthemes of High School Student, Human, Adolescent, Anxiety Disorder, College Students, Computers, Cross-Sectional Studies, and Emotions. Strong relations were also observed between these subthemes as well. To illustrate, studies on High School Student [46], Human [47], Adolescent [48], Anxiety Disorder [49], College Students [50], Computers [51], Cross-Sectional Studies [52], and Emotions [53] support the current study’s findings with regards to the subthemes shown in the Addictive Behavior cluster network.

The analysis also revealed that the main theme of Male (0.89, 0.89) had strong associations with the subthemes of Female, Depression, Reference Values, Risk Factors, School, Sex Factors, Universities, and Video Games. Existing studies on Female [54], Depression [55], Reference Values [56], Risk Factors [57], School [58], Sex Factors [59], Universities [49], and Video Games [60] illustrate the current study’s findings with regards to the Male cluster network.

The main theme of Questionnaire (0.78, 0.78) was found to have associations with Self Control, Social Networking, Parents, Social Network, Social Media Addiction, Cultural Factor, Happiness, and Satisfaction subthemes. Studies on Self-Control [61], Social Networking [62], Parents [46], Social Network [63], Cultural Factor [61], Happiness [64], and Satisfaction [65] illustrate these results with regards to the Questionnaire cluster network.

The analysis revealed that the main theme of Adult (0.56, 0.56) was associated with the subthemes of Psychological Stress, Anxiety, Young Adult, Aggression, Impulse Behavior, Impulsiveness, Culture, and Interpersonal Communication. To illustrate these results, studies on Psychological Stress [54], Anxiety [49], Young Adult [66], Aggression [62], Impulsive Behavior [67], and Culture [68] can be listed as examples.

##### Period 2 (2013–2017)

**Figure 6:**
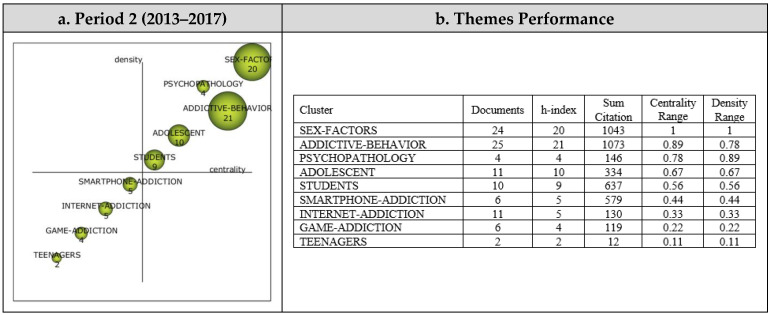
Period 2: (**a**) Strategic diagram and (**b**) performance analysis.

Nine themes emerged from the analysis of the 94 articles reviewed within the scope of Period 2. The strategic diagram and the performance measures for the themes that emerged during the second period are presented in Figure 6.

The most significant theme that emerged during the second period (2013–2017) was Addictive Behavior, represented by 25 published articles. During this period, the themes were gathered in only two regions of the strategic diagram. The Sex Factors, Addictive Behavior, Psychopathology, Adolescent, and Students themes emerged as motor themes. However, the themes of Smartphone Addiction, Internet Addiction, Game Addiction, and Teenagers were among those that were emerging or declining during the second period.

**Figure 7:**
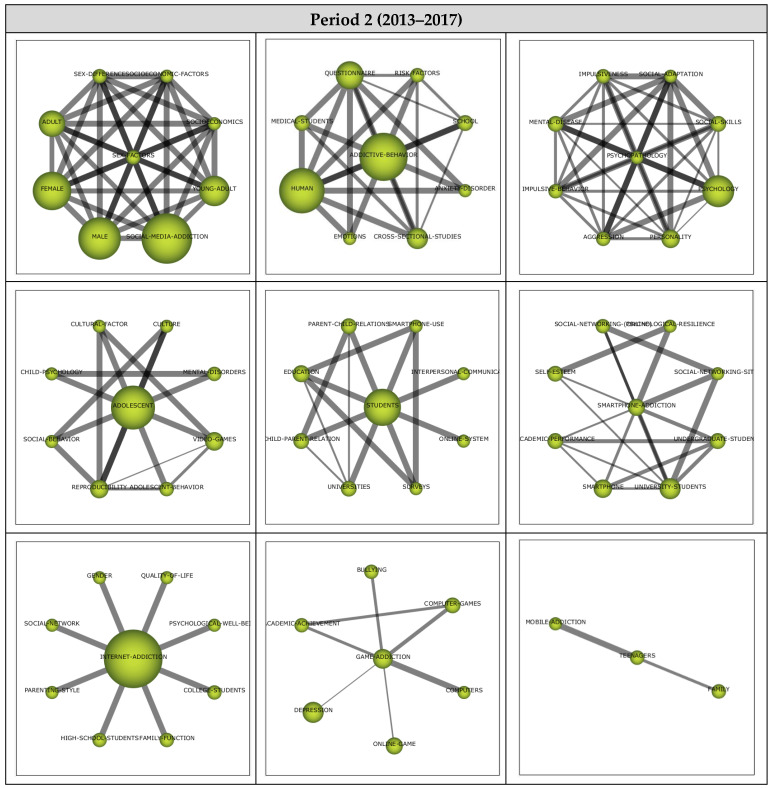
Period 2: Thematic network structures.

The cluster networks (see Figure 7) were examined in order to determine the subthemes associated with the motor themes that emerged during the second period (2013–2017). Accordingly, the main theme of Sex Factor (1, 1) was found to be associated with the subthemes of Socioeconomic Factors, Young Adult, Social Media Addiction, Male, Female, Adult, and Sex Difference. Studies on Socioeconomic Factors [69], Young Adult [70], Social Media Addiction [71], Male [72], Female [73], Adult [74], and Sex Difference [69] are representative of some of the published articles that illustrate these findings with regards to the Sex Factor cluster network.

The main theme of Addictive Behavior (0.89, 0.78) was determined to have strong associations with the Risk Factor, School, Anxiety Disorder, Cross-Sectional Studies, Emotions, Human, Medical Students, and Questionnaire subthemes. To illustrate these results, the studies on Risk Factor [75], School [76], Anxiety Disorder [77], Cross-Sectional Studies [78], Emotions [59], Human [79], Medical-Students [78], and Questionnaire [71] can be listed.

The main theme of Psychopathology was found to be strongly associated with the Social Adaptation, Social Skill, Psychology, Personality, Aggression, Impulsive Behavior, Mental Disease, and Impulsiveness subthemes. These studies on Social Adaptation [62], Social Skill [80], Psychology [74], Personality [79], Aggression [77], Impulsive Behavior [72], Mental Disease [74], and Impulsiveness [81] can be given as illustrative of the findings with regards to the Psychopathology cluster network.

The Students (0.67, 0.67) main theme was found to be associated with the subthemes of Smartphone Use, Interpersonal Communication, Online System, Surveys, Universities, Child–Parent Relation, Education, and Parent–Child Relations. To illustrate these results with regards to the Students cluster network, these studies on Smartphone Use [82], Interpersonal Communication, Online System [83], Surveys [72], Universities [84], Child–Parent [79], Education [85], and Parent–Child Relations [70] may be listed.

The Adolescent (0.56, 0.56) main theme was determined to be associated with the subthemes of Culture, Mental Disorders, Video Games, Adolescent Behavior, Reproducibility, Social Behavior, Child Psychology, and Cultural Factor. These studies on Culture [86], Mental Disorders [87], Video Games [73], Adolescent Behavior [88], Reproducibility [86], Social Behavior [86], Child Psychology [87], and Cultural Factor [73] are some that illustrate these results with regards to the Adolescent cluster network.

##### Period 3 (2018–2022)

**Figure 8:**
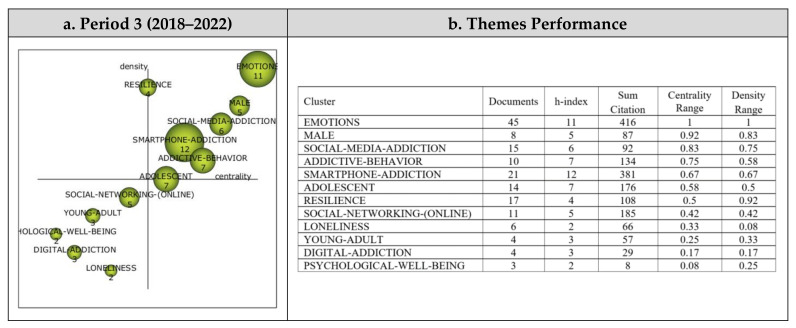
Period 3: (**a**) Strategic diagram and (**b**) performance analysis.

A total of 12 themes emerged from the analysis of the 265 published articles reviewed within the scope of Period 3. The strategic diagram and the performance values for the themes that emerged during the third period are presented in Figure 8.

Among the 12 main themes that emerged during the third period covering the years between 2018 and 2022, the Emotions theme was the most significant, represented by 45 published articles. The themes that emerged during the third period were observed to gather in only two regions of the strategic diagram. The Emotions, Male, Social Media Addiction, Addictive Behavior, Smartphone Addiction, Adolescent, and Resilience themes were found to be the motor themes, whilst Social Networking (Online), Loneliness, Young Adult, Digital Addiction, and Psychological Well-Being were among the emerging/declining themes during the third period.

**Figure 9:**
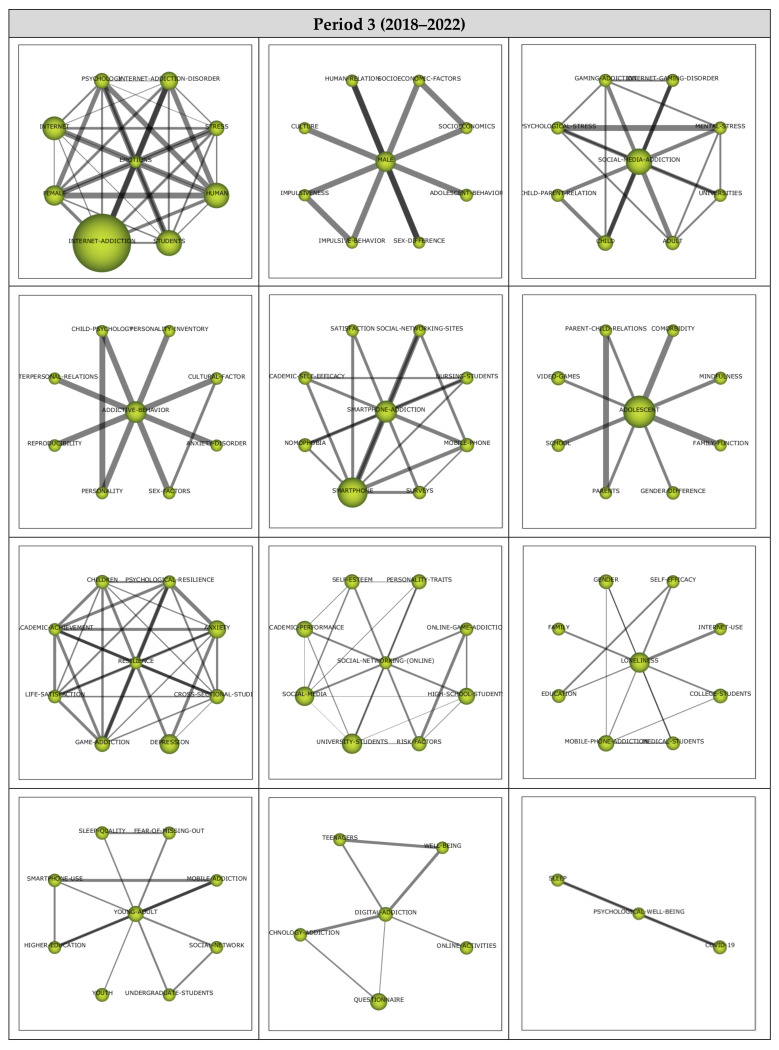
Period 3: Thematic network structures.

Cluster networks (see Figure 9) of the motor themes that emerged during the third period (2018–2022) were examined in order to determine their associated subthemes. The main theme of Emotions (1, 1) was found to have a strong association with the Internet Addiction Disorder, Stress, Human, Students, Internet Addiction, Female, Internet, and Psychology subthemes. Studies on Internet Addiction Disorder [63], Stress [89], Human [90], Students [91], Internet Addiction [92], Female [93], Internet [94], and Psychology [95] are some of the articles published that support these findings with regards to the Emotions cluster network.

The Male (0.92, 0.83) theme, on the other hand, was found to be associated with the subthemes of Socioeconomic Factor, Socioeconomics, Adolescent Behavior, Sex Difference, Impulsive Behavior, and Impulsiveness. These studies on Socioeconomic Factor [96], Socioeconomics [97], Adolescent Behavior [98], Sex Difference [99], and Impulsive Behavior [97] illustrate these findings with regards to the Male cluster network.

The main theme of Social Media Addiction (0.83, 0.75) was found to be associated with an Internet Gaming Disorder, Mental Stress, Universities, Adult, Child, Child–Parent Relation, Psychological Stress, and Gaming Addiction subthemes. These studies on Internet Gaming Disorder [95], Mental Stress [100], Universities [101], Adult [102], Child [103], Child–Parent Relation [104], Psychological Stress [105], and Gaming Addiction [106] illustrate these findings with regards to the Social Media Addiction cluster network.

The main theme of Addictive Behavior (0.75, 0.58) was determined to be associated with the subthemes of Personality Inventory, Cultural Factor, Anxiety Disorder, Sex Factors, Personality, Reproducibility, Interpersonal Relations, and Child Psychology themes. These studies on Personality Inventory [107], Cultural Factor [102], Anxiety Disorder [108], Sex Factors [102], Personality [95], Reproducibility [109], Interpersonal Relations [110], and Child Psychology [98] support the findings with regards to the Addictive Behavior cluster network.

The main theme of Smartphone Addiction (0.67, 0.67) was determined to have associations with the Nursing Students, Mobile Phone, Surveys, Smartphone, Nomophobia, Academic Self-Efficacy, and Satisfaction subthemes. These studies on Nursing Students [111], Mobile Phone [112], Surveys [113], Smartphone [114], Nomophobia [115], Academic Self-Efficacy [116], and Satisfaction [117] support the findings with regards to the Smartphone Addiction cluster network.

The main theme of Resilience (0.5, 0.92) was found to be associated with the subthemes of Psychological Resilience, Anxiety, Cross-Sectional Study, Depression, Game Addiction, Life Satisfaction, Academic Achievement, and Children. Studies on Psychological Resilience [118], Anxiety [119], Cross-Sectional Study [120], Depression [121], Game Addiction [122], Life Satisfaction [123], Academic Achievement [124], and Children [125] support these findings with regards to the Resilience cluster network.

#### 3.2.2. Overlapping Map

**Figure 10:**
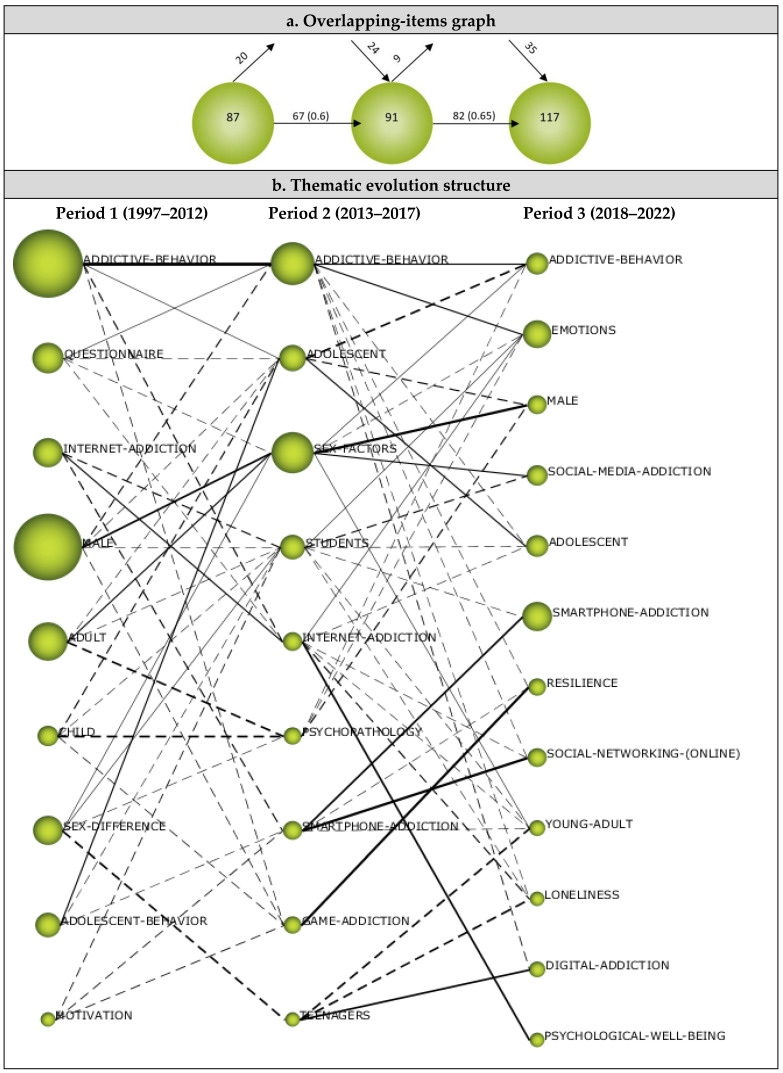
(**a**) Overlapping map and (**b**) thematic evolution map.

An overlapping-items graph shows the number of keywords used within each period, as well as those that were newly appeared, lost, or reused in the subsequent period [126]. When the overlapping map presented in Figure 10a is scrutinized, it can be seen that a total of 87 keywords emerged during the first period of analysis (1997–2012) and that 20 of these keywords were not used during the second period, whilst 67 of them were. During the second period of analysis (2013–2017), on the other hand, a total of 91 keywords emerged, 82 of which were used during the subsequent third period, whilst nine of them were not. The third period (2018–2022) showed a total of 117 keywords having been used, with 24 having been used for the first time during the second period and 35 emerging during the third period. In addition, it was determined that the similarity index remained at a similar level (from 0.6 to 0.65) between the time periods.

The overlapping-items graph (see Figure 10a) revealed that the terminology related to digital addiction has been increasingly consolidated with each passing year and that new keywords have been introduced to the field. Viewed from left to right, the number of keywords shown in Figure 10a increased from 87 during the first period (left) to 117 during the third period (right). This significant increase in the number of keywords used reveals that studies on digital addiction became more diversified over time as the keywords used increased cumulatively. The increase in the number of keywords added during each period indicate that studies on digital addiction were constantly developing, whilst the reserved words indicate that the terms used in this research field were being constantly updated.

#### 3.2.3. Thematic Evolution Structure

The thematic evolution map (see Figure 10b) illustrates the pattern of development in knowledge domains and the relationship between digital addiction-focused research topics over the three periods of analysis. The size of the spheres shown on the map relate to the number of published articles, whilst the thickness of the lines connecting the spheres relate to the correlation between the themes emerged during the analysis periods [32,41].

As shown in the thematic evolution map, nine themes emerged during the first period (1997–2012), which constituted 16.32% of the articles included in the analysis. Six of these themes survived during other periods, and one disappeared without making any further connection. The Addictive Behavior theme that emerged during the first period continued its existence across all three periods and also connected with the Adolescent, Internet Addiction, and Game Addiction themes in the second period. The Questionnaire theme was exchanged with the Addictive Behavior, Sex Factor, and Internet Addiction themes during the second period. The Internet Addiction theme survived during the second period and also connected with the Student and Smartphone themes. The Male theme was exchanged with the Sex Factor theme during the second period and continued as the Male theme during the third period. The Adult theme was observed to evolve into the Sex Factor and Psychopathology themes, the Child theme evolved to become the Adolescent and Sex Factor themes, and the Sex Difference theme to the Sex Factor, Students, and Teenagers themes. The Adolescent Behavior theme was exchanged with Adolescent, Students, and Smartphone Addiction themes, whilst the Motivation theme was exchanged with the Students, Smartphone Addiction, and Game Addiction themes.

During the second period (2013–2017), which constituted 21.91% of the analyzed articles, nine themes emerged. Whilst two of these themes were from the first period, seven of them appeared for the first time during the second period. All of the themes that emerged during the second period were connected with those from either the first or third periods. The Adolescent theme from the second period was shown to have connections with the Addictive Behavior, Male, and Adolescent themes, and the Adolescent theme also continued to exist during the third period. The Sex Factor theme was exchanged with the Male, Social Media Addiction, and Young Adult themes. The Student theme was exchanged with the Emotions, Social Media Addiction, Adolescent, Smartphone Addiction, Young Adult, and Loneliness themes. The Internet Addiction theme evolved into the Emotions, Adolescent, Social Networking (Online), Loneliness, and Psychological Well-Being themes, while the Psychopathology theme evolved into the Addictive Behavior, Emotions, and Male themes. The Smartphone Addiction theme continued to exist in the third period and was also connected with the Social Networking (Online) and Resilience themes. The Game Addiction theme was exchanged with the Resilience theme during the third period, whilst the Teenagers theme was exchanged with the Young Adult, Loneliness, and Digital Addiction themes during the third period.

The third period (2015–2021) was comprised of 61.77% of the analyzed articles, and 12 themes emerged during this period. Among these themes, Addictive Behavior and Smartphone Addiction were transferred from the previous (second) period, whilst 10 new themes emerged during the third period. The themes that emerged for the first time during the third period were Emotions, Male, Social Media Addiction, Adolescent, Resilience, Social Networking (Online), Young Adult, Loneliness, Digital Addiction, and Psychological Well-Being. All the themes that emerged during the third period were observed to be connected with themes from the second period.

## 4. Discussion

The current study investigated the conceptual architecture and intellectual evolution of the digital addiction research field. Using the term digital addiction as an umbrella term incorporating addiction to any type of digital media such as computers, smartphones, video games, and movies, regardless of whether or not an Internet connection is required, the study yielded results that were not only representative of this broader scope of digital addiction as a research field but also delineated the evolving research perspective on the addictive nature of digitalization in contemporary society, moving from Internet addiction to video/online gaming addiction and, more recently, to social media/social networking addiction.

The period-based science mapping analysis in the current study helped reveal the changing research trajectories and topical trends that contributed to the development of the digital addiction knowledge base. To begin with, during the first period of analysis, which constituted research articles published between 1997 and 2012, addictive behavior was found to be the central theme of research. This is not a surprising result considering that research in this area during this initial period was still in its infancy, attempting to establish a theoretical and empirical footing for the investigation of this newer variant of addictive behavior, as opposed to gambling or tobacco smoking addiction or substance abuse [11,127]. Borrowing from the general addiction literature, scholars were inclined to uncover risk factors or outcomes of digital addiction (i.e., initially termed as Internet addiction) as a new type of addictive behavior, which developed in parallel to the digital revolution and the associated accelerating influence on society in general.

The goal of this early research was to describe, diagnose, and treat addiction to Internet-based technologies [128]. More specifically, scholars used previously defined behavioral addiction frameworks to differentiate normal from addictive use of this new media type [129,130]. Digital addiction was in fact distinguished from other behavioral addictions such as gambling or tobacco smoking [131,132], because these technologies have quickly become integral to many aspects of modern daily life, and result not only in harm but numerous benefits as well in people’s everyday lives. In the same vein, establishing valid and reliable criteria to measure addiction to digital media attracted significant debate not only among scholars but also practitioners (e.g., psychologists, psychiatrists, and neuroscientists) [127]. This was a significant concern during this initial period, since the field lacked a solid theoretical and empirical footing. To address this void, Young [133] made the first attempt to develop one of the most widely used questionnaires to measure Internet addiction (the Internet addiction diagnostic questionnaire) based on findings with regards to gambling addiction. All of these developments in the literature may go towards explaining how the Questionnaire emerged as an influential theme during this initial period.

Another reason for the prevalence of the Questionnaire theme could be that many of the published studies that focused on the addictive-behavior theme adopted a cross-sectional study design, and the data for these studies were often gathered by way of administering surveys or questionnaires [134,135]. Cross-sectional studies particularly aim to investigate the prevalence of a disease or pathological problem within a given population and to detect relationships between exposures and outcomes [136]. Thus, they are considered useful for understanding the etiology of health problems and the generation of hypotheses, which therefore may explain the adoption of cross-sectional design for the investigation of digital addiction as a pathological problem. Indeed, closer scrutiny of the prominent themes and subthemes that emerged during the first period indicated a research focus on the cross-sectional investigation of psychological variables underlying digital addiction, such as anxiety, depression, emotions, stress, aggression, self-control, and impulsiveness in different groups such as adults, students, adolescents, and sex (i.e., males vs. females). Yet, cross-sectional designs can be criticized for precluding causal interpretations between exposures and outcomes, as it is impossible to determine the stability of the exposure, to identify whether or not the exposure preceded the outcome(s), and to detect confounding variables [134,137]. As such, it can be logically assumed that research published during this first period had attempted to observe correlations between digital addiction and psychological problems such as loss of control, depression, stress, impulsiveness, or anxiety but were found to be insufficient in determining causal relations between digital addiction and various variables [131]. As Young eloquently explained [132], these studies were unlikely to offer evidence as to whether people became addicted in order to escape feelings of sadness, for instance, or whether the addiction itself was the cause or trigger of such negative feelings. As prompted by Basel et al. [12], it was difficult to determine whether digital addiction was a symptom of an underlying mental health problem or a condition that caused the issue.

During the first period of analysis, two other prominent themes that guided research were found to be males and adults. Research on males was found to have mostly addressed the risk factors for university or school-aged males being addicted to video games in comparison to their female counterparts considering the subthemes associated with the male theme. Earlier evidence that Internet or technology use by males and females had distinctive characteristics [138,139] may have guided scholars towards the comparative investigation of digital addiction between two sexes. With regards to the theme of adults, its associated subthemes revealed a research interest in addiction-related psychological variables such as stress, impulsiveness, depression, anxiety, or aggression in particular to adult or young adult groups.

As for the second period of analysis, which was comprised of the years 2013–2017, the results showed a maintained research interest in addictive behavior and comparison between male and female users, as well as investigation into the psychopathology of addiction. The initial interest of scholars in adult or young adult groups seemingly moved to the addictive impact of digital media on adolescents and students, who were defined as one of the groups most vulnerable to digital addiction [127,139,140]. Internet addiction, which initially appeared as an emerging theme during the first period, was accompanied by smartphone and game addiction during the second period, which indicated the broadening scope of digital addiction research published between 2013 and 2017. Yet, these themes did not take their place among the motor themes, which indicated that scholars were inclined to develop a better understanding into this addictive behavior, especially within adolescent and student populations. Digital technologies have not only changed the way in which adolescents interact, play, communicate, and learn, but also, their excessive use of technology may have alleviated concerns about its potential damage to their mental, physiological, and psychological development [141,142]. It is even emphasized that “desire for digital media is in fact exquisitely aligned with the biology of the teen brain and our evolutionary heritage” and adolescents’ hunger for human connectedness, their appetite for adventure, and desire for information turns digital media into a natural allure for them [143] (p. 127). In light of these findings, future investigations of digital addiction could address behavioral addictions of teenagers or children and particularly focus on newer forms of addictive media such as smartphones or social media as a means of engaging in addictive activities such as chatting, gaming, and shopping [144].

The third period of analysis comprised research articles that were published between 2018 and the first quarter of 2022. Social media addiction and smartphone addiction were two prominent themes noted during the third period. Recent evidence has shown that more than 4 billion people are now users of the Internet, mobile phones, and social media [10,145]. Given the widespread use of smartphones and social media during the past decade for dealing with the demands of work, socialization, and for pleasure, smartphone or social media dependency has prominently been added to academic discussions regarding digital addiction [5,131]. As Hoehe and Thibaut [1] recently pointed out, there is increasing public concern that people are now interacting more through their digital devices than they are in person and that smartphones are changing the social fabric of communities through decreased human capacity for empathy, introspection, creativity, and productivity. As the current study’s results indicate, this has also been notably of concern to scholars, having guided research through the impact of smartphone or social media addiction over the past 5 years. This finding was also supported by Meng et al.’s [10] study in that research on social media and smartphone addiction peaked during 2020–2021. One particular reason for this increase could be the influence of the COVID-19 pandemic, which increased the risks for digital addiction due to school and university-aged students, as well as many adults, forcibly having stayed at home under quarantine conditions, lockdowns, and a general worldwide extended period of remote working/schooling. The pandemic not only accelerated peoples’ exposure to the Internet and digital media but also induced psychological problems, which, in return, increased the risks for increased digital addiction [10,17,146].

The current study also revealed that research during the third period focused on the theme of resilience. Resilience is broadly defined as a person’s capability to adapt to adverse situations in a positive manner, and previous research showed that the resilience of adolescents or students could decrease the likelihood of their developing behavioral addictions such as alcohol or gambling addiction [139,147]. This may have boosted research interest in the relationship between digital addiction and resilience. A focus on resilience is also significant, since it might suggest a different perspective in the investigation of digital addiction, in that previous studies mostly focused on the risk factors or outcomes of digital addiction, whilst the focus of resilience research is more on the protective factors that can reduce addiction behaviors and/or their negative consequences [147,148]. As suggested previously (e.g., [144,149]), the knowledge of risk factors for digital addiction would not suffice for a thorough understanding of digital addiction, but the knowledge of protective factors should also be developed in order to help those at risk of addiction both to stay safe despite risk experiences and to thrive or cope with negative consequences in the case of addiction. Therefore, future studies focusing on protective factors such as resilience in the face of addiction to different types of digital media could contribute greatly to the theory of digital addiction.

Another finding that deserves particular attention was that digital addiction was only observed as a standalone term during the third period and that it was an emerging theme. This may indicate the conceptual recognizance of digital addiction as an overarching term to refer to addiction to any type of digital media, yet future studies are still warranted for its further development. Given that the variety of digital technologies is constantly increasing, it is likely that several types of digital addiction have yet to emerge, but as Lin et al. [131] suggested, handling all these types of addiction as a generalized pathology would help to develop a broader and overarching framework for the prevention, diagnosis, and treatment of digital addiction.

## 5. Conclusions

The current study offered a different perspective into the digital addiction research field through revealing the intellectual and conceptual evolution of this knowledge base. The findings not only determined the current status of this field of study but also offered a guideline for future studies through revealing the insufficiently illuminated or underdeveloped aspects of digital addiction as a field of study. Thus, the current study contributed to the understanding of digital addiction as the pathology of the current and, most probably, the coming era. Another strength of the study was its coverage of research on not only Internet addiction but on any type of addiction resulting from the excessive use of various novel technologies, which enabled the development of a broader and cumulative understanding into the investigation of these affiliated types of addiction.

The current study also presents certain limitations. First, the study may have missed some of the research published on digital addiction despite the wide coverage of journals and articles listed on the Scopus database, and the inclusion of a larger scope of research thanks to the co-word analysis. More specifically, the current study addressed articles with a particular focus on ‘addiction’ and, thus, used ‘addiction’ as the primary keyword during the data search process while excluding ‘problematic use’ of digital media/devices. Despite its increasing popularity in the literature, digital addiction has not yet been included in the Diagnostic and Statistical Manual (DSM) or in the International Classification of Diseases (ICD), so there is the likelihood that the present analysis might have missed some research that addressed such behaviors as problematic use of digital media. Furthermore, despite being a review study, the current study did not present the findings of previous research but only mapped the scientific evolution of the research field. Therefore, future reviews of digital addiction could be conducted using different methods such as a meta-analysis, meta-synthesis, or systematic literature review. Similarly, future science mapping studies could help to observe the evolution of the main themes emerged during the third period of the current study’s analysis (2018–2022) over the next couple of years.

## Figures and Tables

**Table 1:** Inclusion/exclusion criteria.

Criteria	Included	Excluded	Rationale
***Language***	English	Other languages	Internationally used and researchers’ ability to understand
***Document type***	Journal articles	Books, book chapters, conference proceedings	Targeting of refereed, high-quality publications
***Database***	Scopus	Other databases (e.g., Web of Science, PubMed, etc.)	Broader coverage of journals/articles

**Table 2:** Top 10 authors most cited in digital addiction.

Rank	Author	TC *	TP	h-İndex
1	Young, Kimberly, S.	3219	5	29
2	Ko, Chihhung	1072	7	52
3	Yen, Chengfang	1070	6	56
4	Yen, J. Y.	1070	6	45
5	Leung, Louis	918	5	35
6	Lemmens, Jeroen, S.	592	1	13
7	Peter, Jochen	592	1	53
8	Valkenburg, Patti, M.	592	1	64
9	Chen, Chengchung	486	2	31
10	Hawi, Nazir, S.	483	5	14

* TC: total citations; TP: total publications. Data retrieved from Scopus, 9 July 2022.

**Table 3:** Top 10 journals in terms of the number of publications on digital addiction.

Rank	Journal Name	TP *	TC	SJR	Scopus Quartile
1	*Cyberpsychology, Behavior, and Social Networking*	44	2339	1.15	Q1
2	*Children and Youth Services Review*	25	546	0.80	Q1
3	*Cyberpsychology and Behavior*	25	6852	n/a	n/a
4	*Turkish Online Journal of Educational Technology*	10	126	n/a	n/a
5	*Behavior and Information Technology*	8	403	0.70	Q1
6	*Social Science Computer Review*	8	560	1.50	Q1
7	*Computers and Education*	7	769	3.68	Q1
8	*Universal Journal of Educational Research*	7	37	n/a	n/a
9	*International Journal of Adolescence and Youth*	7	57	0.85	Q1
10	*Contemporary Educational Technology*	6	38	0.72	Q1

* TP: Total publications; TC: Total citations; SJR: Scientific Journal Ranking Data retrieved from Scopus, 9 July 2022.

**Table 4:** Top 10 articles cited in digital addiction.

Rank	Article Name	Journal Name	Author(s)	Year	TC *
1	Internet addiction: The emergence of a new clinical disorder	*Cyberpsychology and Behavior*	Young, K. S.	1998	2608
2	Development and validation of a game addiction scale for adolescents	*Media Psychology*	Lemmens, J. S.; Valkenburg, P. M.; Peter, J.	2009	592
3	Internet addiction: A new clinical phenomenon and its consequences	*American Behavioral Scientist*	Young, K. S.	2004	578
4	Internet addiction, usage, gratification, and pleasure experience: The Taiwan college students’ case	*Computers and Education*	Chou, C.; Hsiao, M.-C.	2000	410
5	Shyness and locus of control as predictors of Internet addiction and Internet use	*Cyberpsychology and Behavior*	Chak, K.; Leung, L.	2004	402
6	Internet addiction on campus: The vulnerability of college students	*Cyberpsychology and Behavior*	Kandell, J. J.	1998	381
7	Online gaming addiction: The role of sensation seeking, self-control, neuroticism, aggression, state anxiety, and trait anxiety	*Cyberpsychology, Behavior, and Social Networking*	Mehroof, M.; Griffiths, M.D.	2010	361
8	Prevalence of Internet addiction and correlations with family factors among South Korean adolescents	*Adolescence*	Park, S.-K.; Kim, J.-Y.; Cho, C.-B.	2008	303
9	Factors Associated with Internet Addiction among Adolescents	*Cyberpsychology and Behavior*	Lam, L.-T.; Peng, Z.-W.; Mai, J.-C.; Jing, J.	2009	301
10	Family factors of Internet addiction and substance use experience in Taiwanese adolescents	*Cyberpsychology and Behavior*	Yen, J.-Y.; Yen C.-F.; Chen, C.-C.; Chen, S.-H.; Ko, C.-H.	2007	287

(*) Data retrieved from Scopus, 9 July 2022.

**Table 5:** Top 10 countries with most publications in digital addiction.

Rank	Country	TP *	TC
1	Turkey	91	1420
2	China	55	2153
3	United States	49	4877
4	South Korea	39	1230
5	Taiwan	37	2962
6	Hong Kong	24	1675
7	Russian Federation	21	98
8	Iran	18	219
9	Malaysia	17	92
10	India	13	355

(*) Data retrieved from Scopus, 9 July 2022.

## Data Availability

Data used are publicly available; no identifying information was collected or included. All the data used in this research was accessed through the Scopus database.
